# Epidemiology and risk analysis of Powassan virus infection, New York state, USA, 2013–2023

**DOI:** 10.1017/S0950268826101113

**Published:** 2026-02-02

**Authors:** Angelina R. Varon, Melissa A. Prusinski, Collin O’Connor, Joseph G. Maffei, Lindsay Tomaszek, Jessica Stout, Anne F. Payne, Alan P. Dupuis, Alexander T. Ciota, Kyle Carson, Lindsey A. Jones, Kelly Howard, William T. Lee, Jennifer White

**Affiliations:** 1 Association of Public Health Laboratories, USA; 2 Bureau of Communicable Disease Control, New York State Department of Health, USA; 3 Bouvé College of Health Sciences, Northeastern University – Boston Campus, USA; 4 Department of Geography, State University of New York at Buffalo, USA; 5 The Arbovirus Laboratory, New York State Department of Health, Wadsworth Center, USA; 6 Diagnostic Immunology Laboratory, New York State Department of Health, Wadsworth Center, USA

**Keywords:** epidemiology, *Ixodes scapularis*, Powassan encephalitis, Powassan virus, tick-borne diseases

## Abstract

Powassan virus (POWV), a tick-borne flavivirus, is an emerging public health threat in the United States. In New York State (NYS), incidence of human POWV infection has increased in recent years. We describe the epidemiology of confirmed and probable POWV infection cases reported in NYS from 2013 to 2023. A total of 44 human cases were reported over the study period, with the highest incidence rates in Columbia and Putnam counties. Most cases occurred in White, non-Hispanic males over age 50. Hospitalization was reported in 91% of cases, and 11% were fatal. Human case data and tick surveillance results were analysed to assess spatiotemporal patterns of disease emergence. Spatial analysis revealed clustering of human cases in the Capital and Metropolitan regions of NYS. The prevalence of POWV in adult tick populations increased significantly statewide, and entomological risk was positively but modestly correlated to disease incidence at the ZIP code level. These findings suggest that POWV infection is emerging in geographically concentrated areas of NYS, highlighting the need for enhanced surveillance and targeted prevention efforts in high-risk regions.

## Key results

- From 2013 to 2023, POWV infection incidence in New York State was highest in southeastern counties, particularly Columbia and Putnam.
- The majority of reported cases occurred in White, non-Hispanic males aged ≥50 years; hospitalization was reported in 91% of cases, and 11% were fatal.
- Prevalence of POWV in *I. scapularis* populations increased over the study period statewide (p < 0.05), with the highest increases among adult ticks in the Capital Region (p < 0.002).
- Spatial clustering of human cases corresponded to areas with elevated Entomologic Risk Index (ERI).
- Adult ERI showed a significant association with human POWV incidence at the ZIP code level (p < 2.2e-16, Spearman’s *p* = 0.195), underscoring the importance of surveillance in identifying high-risk areas for targeted public health interventions.

## Introduction

Powassan virus (POWV) is an emerging tick-borne flavivirus that causes long-term neurological sequelae in about half of reported clinical cases, and fatality in about 10 % of cases classified as neuroinvasive [1–3]. First identified in Powassan, ON, Canada, POWV was isolated from the brain tissue of a paediatric patient with fatal encephalitis in 1958 [4]. The first human case in the United States (US) was later identified in New Jersey in 1970 [5]. POWV is the only tick-borne flavivirus found in North America and is presently endemic to North America and Northeastern Russia [6].

There are two distinct genetic lineages of POWV, which can cause infection in humans: lineage I, prototype Powassan virus (POWV-1), and lineage II, Deer Tick virus (DTV) [7]. While POWV-1 was isolated from the first Powassan case in North America, DTV was not identified in ticks in North America until 1995 [7]. The two POWV lineages, while genetically and ecologically distinct, cannot be serologically distinguished [8].

POWV lineage I and II each have unique enzootic cycles but are both maintained in the environment between Ixodid ticks and various vertebrate hosts. POWV-1 is principally sustained between *Ixodes cookei* ticks and groundhogs (*Marmota monax*) and mustelids. It is also cycled between *Ixodes marxi* ticks and arboreal squirrels [9]. DTV is thought to be maintained between *I. scapularis* ticks and white-footed mice (*Peromyscus leucopus)* [7, 9]. Recent evidence also suggests that shrews may serve as a reservoir of DTV for host-seeking *I. scapularis* ticks [10]. *I. scapularis* is a generalist which feeds on a wide variety of hosts, including humans [11, 12]. As such, *I. scapularis* transmits a variety of pathogens across its geographic range and is likely the primary vector of POWV infection in humans in the US [6].

Transmission of pathogens from *I. scapularis* ticks occurs during blood feeding [13]. While transmission of other pathogens from *I. scapularis*, such as *Borrelia burgdorferi* (causative agent of Lyme disease) and *Babesia microti* (causative agent of babesiosis), take a minimum of 36 h of tick attachment, POWV has been demonstrated experimentally to transmit in as little as 15 min of tick feeding time [14]. In humans, POWV infection has occurred after less than 6 h of tick attachment [13]. The shortened transmission window of POWV heightens the risk of human infection when compared with other tick-borne pathogens because feeding ticks often go unnoticed and public health messaging emphasizing “daily tick checks” may prove ineffective at preventing virus transmission.

Historically, POWV morbidity and mortality in the US has been low despite its rapid transmission rate and high case-fatality rate. However, cases of POWV infection have increased considerably across the US over the last two decades, likely due to a combination of increased disease diagnostic capacity, surveillance efforts, and increases in vector distribution and density driving disease emergence [3]. POWV human infection is also suspected to be more common than reported case data indicate, as subclinical or mild cases of POWV are rarely reported [8]. In the US, human POWV cases are concentrated in the Northeast and Upper Midwest [3]. New York State (NYS) is among several states with the highest number of reported cases of POWV human infection in the country, including Minnesota and Wisconsin in the Midwest, and Massachusetts in the Northeast. Reported POWV cases in these four states accounted for nearly 70% of all reported cases nationwide from 2004 to 2023 [3]. In the years from 2013 to 2023, 291 human cases of POWV were reported nationally, 15% of which were from NYS (n = 44) [3]. The goals of this study were to better describe the epidemiology of POWV infection in NYS and to potentially identify populations at increased risk of exposure to POWV. Tick-borne pathogen surveillance data from the NYS Department of Health (NYSDOH) was also analysed to assess the presence and distribution of POWV in tick populations across NYS to elucidate geospatial risk for POWV infection.

## Methods

### Powassan virus cases

We conducted a review of all human cases of POWV infection that were reported to the NYSDOH during 2013–2023. POWV infections and arboviral encephalitis are reportable conditions under NYS public health law [15]. Suspect cases are reported directly by medical providers and electronically through positive commercial laboratory reports. Local county health departments conduct case investigations to determine case status based on the prevailing national surveillance case definition at time of diagnosis [16]. Case data obtained from investigation efforts were entered and tracked in the NYSDOH Communicable Disease Electronic Surveillance System. Both confirmed and probable cases were included in this study. Case demographic data were summarized in Microsoft Excel version 16.93.1 using descriptive statistics, mean and SD for continuous variables, and counts and percentages for categorical variables.

### Tick collection and testing

Host-seeking *I. scapularis* were collected from public lands across NYS from 2013 to 2023 using standardized dragging and flagging surveys as previously described [17]. Collection sites were chosen based on tick habitat suitability and potential for human exposure to ticks (*e.g.* presence of understory vegetation and hiking trails) or were epidemiologically linked to cases of POWV infection. *I. scapularis* larvae and nymphs were primarily collected during periods of peak larval (August) and nymphal (May–July) activity by dragging a 1m^2^ piece of white flannel through leaf litter and low brush. *I. scapularis* adults were primarily collected during periods of peak adult (October–November) activity by flagging a 1m^2^ piece of white canvas along understory vegetation up to 1 m high. Ticks were stored at 4 °C until identified using dichotomous keys and sorted into pools by collection site and date, species, and developmental stage [18]. Pools of up to ten ticks each were stored at −80 C until screened by real-time RT-PCR for the presence of POWV-1 and DTV as previously detailed [19].

### Data analysis

POWV case reports meeting criteria for inclusion were analysed using SAS 9.4. POWV infection cases were mapped using RStudio 2024.04.2 + 764 and QGIS 3.34 according to ZIP code tabulation area (ZCTA) using ZIP code of patient residence and 2018 American Community Survey 5-year estimates of population and shapefile [20]. Spatial autocorrelation of POWV infection cases at the ZCTA level was determined using the global and local Moran’s *I* statistic. Moran’s *I* k-nearest neighbour value was estimated *a priori* as the average number of ZCTAs per county in NY (k = 29). Local Moran’s *I* test identified clusters of POWV as statistically significant using α of 0.05.

**Figure 1.**
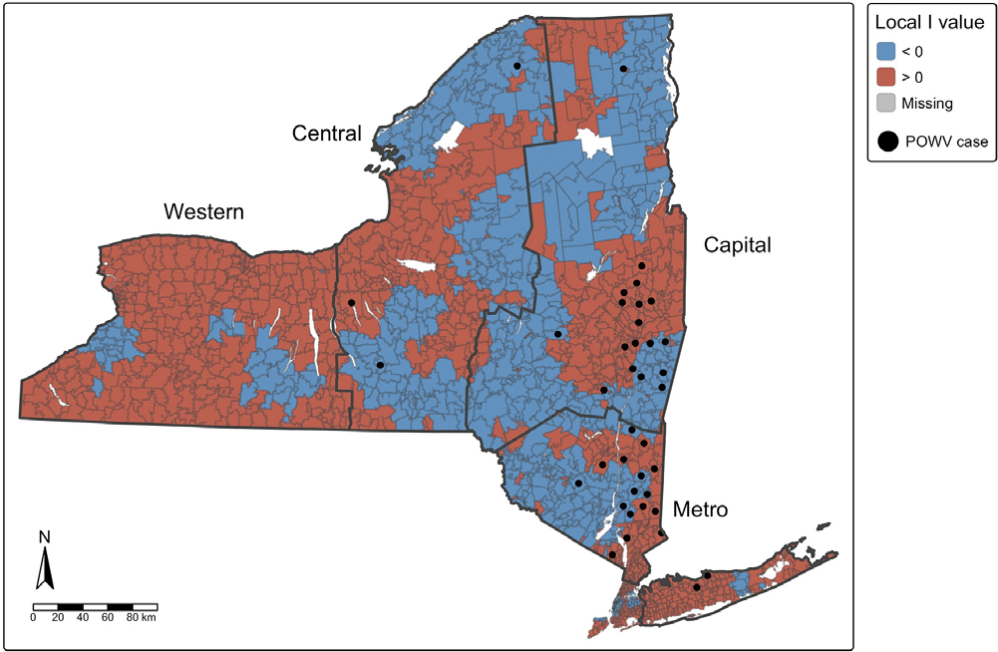
Spatial autocorrelation of cases of Powassan virus infection (Moran’s I), with human cases overlaid by ZIPCode Tabulation Area (ZCTA), New York State, 2013–2023. Positive values (red) indicate ZCTAs where highrisk areas are surrounded by other high-risk areas (high-high clustering), while negative values (blue) indicate ZCTAs where low-risk areas are surrounded by other low-risk areas (low-low clustering). ZCTAs where high-risk areas are adjacent to low-risk areas (or vice versa) are considered spatial outliers.

Tick collection and pathogen prevalence, statistical analyses, and data visualizations were achieved with Microsoft Excel, R studio 2024.04.2 + 764 and QGIS 3.34. Pathogen prevalence was calculated as the minimum infection rate, or the number of pools of *I. scapularis* ticks testing positive for POWV divided by the total number of ticks tested at the site level and for each of four NYS regions (Capital, Central, Metro, and Western) (Figure 1). A linear regression model was built to estimate temporal changes in pathogen prevalence in *I. scapularis* populations over the study period (α = 0.05). Risk of exposure to POWV was estimated using an entomologic risk index (ERI), a measure of the population density of pathogen-carrying ticks [21]. ERI was calculated separately for nymphal and adult ticks as the product of tick population density (ticks per 1,000 m^2^ sampled) and POWV prevalence at each collection site. ZCTA-level ERI was calculated as the average ERI of all sites within the ZCTA for each tick developmental stage over the study period. Correlation of POWV infection incidence and ERI over the study period was assessed at the ZCTA level using Spearman’s rank correlation.

## Results

### Powassan virus epidemiology

A total of 44 cases of POWV were reported in NYS from 2013 to 2023, excluding data from New York City, which conducts independent jurisdictional vector and epidemiological surveillance and reporting for the 5 New York City boroughs. Over the study period, a median of three cases/year were reported across NYS (range: 1–9 cases/year) (Table 1). County-level incidence rates over the 11-year study period were highest in two counties in southeastern NYS: Columbia County (0.59 cases/100,000 person-years at risk) and Putnam County (0.46 cases/100,000 person-years at risk) (Table 2). The statewide incidence was 0.02 cases/100,000 person-years at risk over the study period.

**Table 1.** Demographic and epidemiological characteristics of reported cases of Powassan virus infection in New York State, 2013–2023

Characteristic	Cases *n*(%) *n = 44*
Case status	
Confirmed	40 (90.91)
Probable	4 (9.09)
Sex	
Male	26 (59.09)
Female	18 (40.91)
Age group (years)	
0–9	9 (20.45)
10–19	2 (4.55)
20–29	1 (2.27)
30–39	1 (2.27)
40–49	2 (4.55)
50–59	5 (11.36)
60–69	11 (25.00)
70–79	7 (15.91)
80+	6 (13.64)
Age (years)	
Mean (SD)	50.14 (28.83)
Median [Min, Max]	61.5 [0.063, 91]
Race	
Black	1 (2.27)
White	37 (84.09)
Unknown	6 (13.64)
Ethnicity	
Non-Hispanic	35 (79.55)
Hispanic	
Unknown	9 (20.45)
Clinical classification	
Neuroinvasive	39 (88.64)
Non-neuroinvasive	5 (11.36)
Outcome	
Alive	36 (81.82)
Deceased	5 (11.36)
Unknown	3 (6.82)

**Table 2.** Number of reported cases of Powassan virus infection and incidence rates per 100,000 population by county, New York, 2013–2023

County	Cases	Incidence rate per 100,000 population
Albany	3	0.09
Cayuga	1	0.12
Clinton	1	0.11
Columbia	4	0.60
Dutchess	9	0.28
Greene	2	0.38
Putnam	5	0.46
Rensselaer	3	0.17
Rockland	1	0.03
Saratoga	6	0.24
Schoharie	1	0.29
St. Lawrence	1	0.08
Suffolk	2	0.01
Tompkins	1	0.09
Ulster	1	0.05
Westchester	3	0.03
State Total	44	0.02

**Figure 2.**
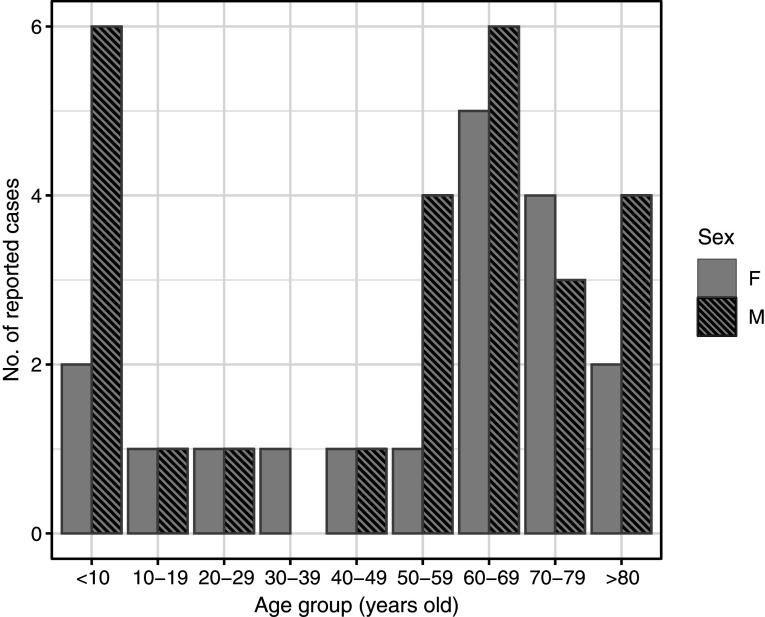
Number of reported cases of Powassan virus infection by age group and sex, New York, 2013–2023 (N = 44).

**Figure 3.**
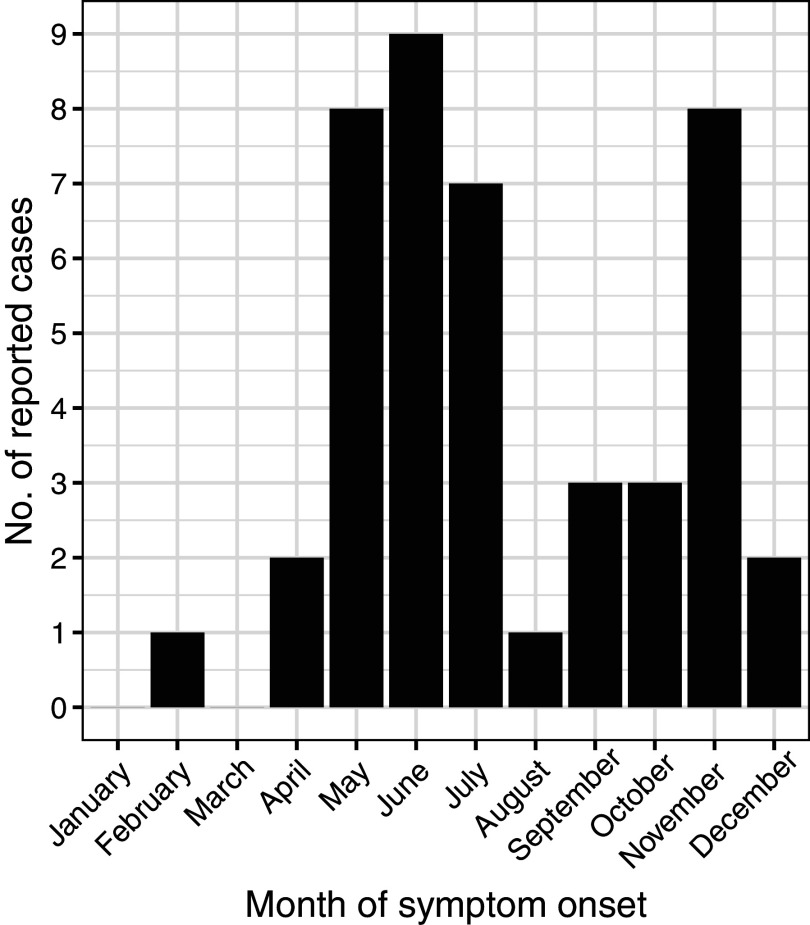
Number of reported cases of Powassan virus infection by month of symptom onset, New York, 2013–2023 (N = 44).

Cases of POWV infection were most common among males and those who identified as White and non-Hispanic (Table 1). Patients over the age of 50 accounted for 65.9% of cases, while those under age ten accounted for 20.5% of cases (Figure 2). Notably, two cases were infants, one aged 1 month, and the other 23 days old. Of the 44 patients diagnosed with confirmed or probable POWV infection, 90.9% were hospitalized. Five (11.4%) patients died and the outcome for three patients (6.8%) is unknown. The most common clinical symptom among patients with neuroinvasive POWV infection was encephalitis, followed by a broad range of other neuroinvasive symptoms and meningitis (Table 3). Acute flaccid paralysis and respiratory failure were the least common clinical syndromes among reported cases. All (100%) patients with non-neuroinvasive POWV infection reported symptoms of febrile illness. Symptom onset occurred most often in patients between May–July and in November (Figure 3).

**Table 3.** Reported clinical signs and symptoms among 44 persons with Powassan virus infection, New York, 2013–2023

Clinical classification	Confirmed	Probable	Total
Neuroinvasive	36	3	39
Acute flaccid paralysis, respiratory failure	1		1
Encephalitis	16	1	17
Encephalitis/Meningitis		1	1
Meningitis	8		8
Other neuroinvasive presentation	10	1	11
Other neuroinvasive presentation; respiratory arrest	1		1
Non-neuroinvasive	4	1	5
Febrile illness	4	1	5

*Note*: Likely incomplete symptomologies. Includes all available information provided by the NYSDOH Communicable Disease Electronic Surveillance System.

### Ecological prevalence of Powassan virus

A total of 58,574 adult, 18,462 nymphal, and 30,588 larval *I. scapularis* were tested in pools for the presence of POWV (POW-1 and DTV) from 2013 to 2023. A total of 292 adult pools (0.50%), 42 nymphal pools (0.23%), and five larval pools (0.02%) tested positive for POWV. Statewide prevalence of POWV increased in larval, nymphal, and adult *I. scapularis* populations over the study period (Table 4). POWV prevalence in larval *I. scapularis* increased in only the Capital Region, with an overall statewide increase from 0.00% in 2013 to 0.03% in 2023. POWV prevalence in nymphal *I. scapularis* increased in both the Capital and Metropolitan regions, with an average yearly statewide increase of 0.0156% over the study period. A linear regression analysis showed that POWV prevalence in adult *I. scapularis* increased significantly (p < 0.05) statewide over the study period, with increases in prevalence in the Capital, Central, and Western regions. In the Capital Region, linear regression revealed a statistically significant (p < 0.002) increase in POWV prevalence in adult *I. scapularis*, from 0.00% in 2013 to 0.63% in 2023. Site-level ERI ranged from 0 to 8 in nymphs and from 0 to 22.2 in adult *I. scapularis.*

**Table 4.** Prevalence of Powassan virus in larvae, nymph, and adult *Ixodes scapularis* by New York State region, 2013–2023

Region	Life stage	Number of ticks tested (per cent positive for Powassan virus)										
		2013	2014	2015	2016	2017	2018	2019	2020	2021	2022	2023
Capital	Larvae	0 (0.00)	0 (0.00)	0 (0.00)	0 (0.00)	3,734 (0.00)	4,139 (0.05)	4,738 (0.00)	3,345 (0.03)	1753 (0.00)	1705 (0.00)	5,986 (0.03)
	Nymphs	179 (0.00)	81 (0.00)	143 (0.00)	96 (0.00)	856 (0.58)	347 (0.00)	1,629 (0.06)	240 (0.42)	1,612 (0.31)	665 (0.75)	1,530 (0.13)
	Adults	710 (0.00)	2050 (0.24)	1,359 (0.00)	974 (0.31)	3,109 (0.64)	1,562 (0.58)	7,499 (0.52)	1,586 (0.69)	4,737 (0.72)	3,393 (0.56)	4,774 (0.63)
Central	Larvae	0 (0.00)	0 (0.00)	0 (0.00)	0 (0.00)	975 (0.00)	37 (0.00)	79 (0.00)	33 (0.00)	1,416 (0.00)	27 (0.00)	55 (0.00)
	Nymphs	0 (0.00)	0 (0.00)	77 (0.00)	221 (0.00)	490 (0.00)	10 (0.00)	72 (0.00)	25 (0.00)	101 (0.00)	14 (0.00)	294 (0.00)
	Adults	445 (0.00)	647 (0.00)	259 (0.00)	1,357 (0.00)	388 (0.00)	355 (0.56)	397 (0.50)	744 (0.13)	407 (0.00)	602 (0.00)	191 (0.00)
Metro	Larvae	0 (0.00)	0 (0.00)	0 (0.00)	0 (0.00)	0 (0.00)	0 (0.00)	0 (0.00)	118 (0.00)	187 (0.00)	0 (0.00)	0 (0.00)
	Nymphs	416 (0.48)	300 (0.00)	350 (0.00)	109 (0.00)	400 (0.75)	731 (0.96)	838 (0.24)	614 (0.65)	1,596 (0.06)	946 (0.32)	970 (0.10)
	Adults	352 (0.57	419 (2.39)	422 (0.47)	902 (0.89)	1,461 (1.23)	899 (1.56)	906 (0.88)	891 (2.24)	1,479 (0.61)	1,225 (1.39)	1,022 (0.74)
Western	Larvae	0 (0.00)	0 (0.00)	0 (0.00)	0 (0.00)	70 (0.00)	47 (0.00)	361 (0.00)	435 (0.00)	416 (0.00)	158 (0.00)	774 (0.00)
	Nymphs	132 (0.00)	150 (0.00)	233 (0.00)	4 (0.00)	99 (0.00)	20 (0.00)	335 (0.00)	233 (0.00)	578 (0.00)	410 (0.00)	316 (0.00)
	Adults	430 (0.00)	281 (0.00)	672 (0.00)	722 (0.00)	765 (0.00)	603 (0.00)	1723 (0.00)	1,608 (0.00)	1,407 (0.00)	1,471 (0.00)	1,314 (0.08)
New York State (excl. NYC)	Larvae	0 (0.00)	0 (0.00)	0 (0.00)	0 (0.00)	4,779 (0.00)	4,223 (0.05)	5,178 (0.00)	3,931 (0.03)	3,772 (0.00)	1890 (0.00)	6,815 (0.03)
	Nymphs	727 (0.28)	531 (0.00)	803 (0.00)	430 (0.00)	1845 (0.43)	1,108 (0.63)	2,874 (0.10)	1,112 (0.45)	3,887 (0.15)	2035 (0.39)	3,110 (0.10)
	Adults	1937 (0.10)	3,397 (0.44)	2,712 (0.07)	3,955 (0.28)	5,723 (0.66)	3,419 (0.73)	10,525 (0.47)	4,829 (0.66)	8,030 (0.54)	6,691 (0.54)	7,356 (0.53)

### Spatial analysis

Spatial analyses were conducted at the ZCTA level, which provides sufficient geographic detail for Moran’s *I* analyses given the small number of POWV infection cases. Regions are included in spatial figures to summarize broader geographic trends across NYS. Counties were not included in spatial analyses and are presented solely for descriptive purposes when reporting incidence rates (Table 2).

Global Moran’s *I* analysis identified statistically significant positive spatial autocorrelation among cases of human POWV infection across NYS ZCTAs (Moran’s *I* = 0.0618, standard deviation = 10.95, *p* < 2.2 × 10^−16^), indicating that POWV cases tended to cluster geographically. Local Moran’s *I* analysis further demonstrated distinct spatial patterns, particularly in the Capital and Metropolitan regions (Figure 1). Out of 1,793 ZCTAs in NYS, 103 (5.7%) had statistically significant local Moran’s *I* values (p < 0.05), supporting evidence of spatial clustering.

**Figure 4.**
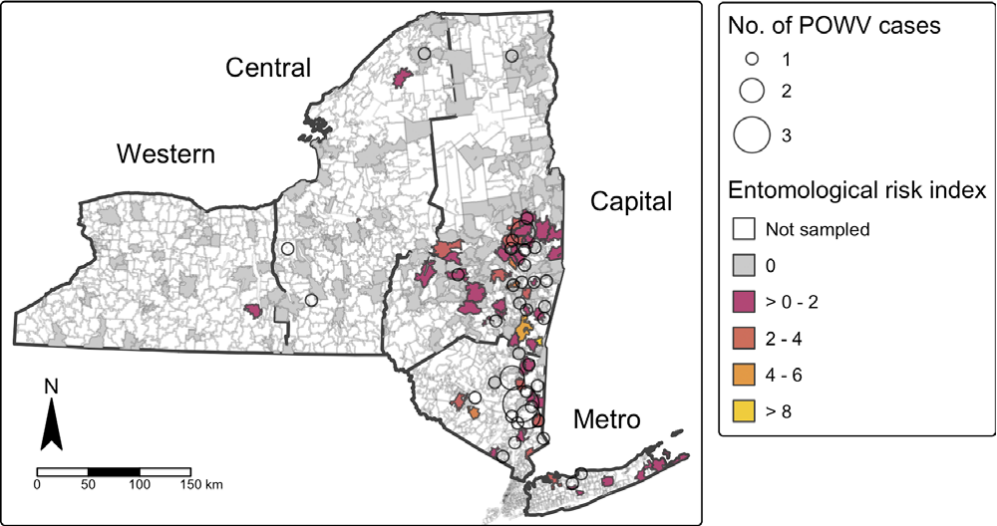
Powassan virus encephalitis cases by zip code tabulation area and *Ixodes scapularis* entomologic risk index (ERI).

While statistical significance at the local level was limited due to minimal data and low number of POWV cases, clear spatial trends are apparent. When overlaid with entomological surveillance data, the spatial distribution of human cases corresponded closely with areas of elevated adult tick entomological risk index (ERI) (Figure 4). Most human cases occurred in ZCTAs where ERI values fell within the range of 2–4 or > 4. Adult ERI was significantly associated with the incidence of POWV infection at the ZCTA level over the study period (p < 2.2e-16), suggesting that entomological surveillance may be a useful predictor of human POWV infection risk, although the correlation coefficient (Spearman’s *p* = 0.195) indicates a modest effect size.

## Discussion

The basic epidemiological characteristics of POWV infections in NYS are consistent with national POWV infection reports, and comparable to other tick-borne diseases transmitted by *I. scapularis.* POWV infection, like Lyme disease, babesiosis, and anaplasmosis, disproportionately affects White individuals and males, likely due to differences in behaviour and exposure risk rather than regional population composition [22–24]. Both the hospitalization and case fatality rates of POWV infection in NYS over the study period were slightly lower (90.9% and 11.4%, respectively) than the national rate (92.1% and 13.4%, respectively) over the same time period [25].

The age distribution of POWV infection is bimodal, with peaks occurring in those under ten and over 60 years of age [25]. The higher risk for individuals under ten may be attributed to factors such as increased time spent in grassy or wooded tick habitats as well as reliance on others for tick-checks. Those aged over 60 may face greater risk of symptomatic POWV infection due to decreased immunocompetence and increased likelihood of engagement in outdoor leisure activities like gardening and dog walking [26]. Like POWV infection, Lyme disease also shows a bimodal age distribution, with peaks in those aged 5–9 and 50–55 years. In contrast, anaplasmosis and babesiosis show unimodal peaks in older adults, likely due to subclinical or asymptomatic expression of these diseases in paediatric populations [22–24].

POWV infection is the only encephalitic tick-borne disease endemic in North America, thus, its symptoms differ from those of other nationally endemic tick-borne diseases [6]. Encephalitis is the most commonly reported clinical syndrome in patients with neuroinvasive POWV infection, a manifestation seen in less than 1 % of Lyme disease cases and not typically reported in cases of babesiosis or anaplasmosis [20–22]. Both the hospitalization and case fatality rates for POWV infection in NYS (90.9% and 11.36%, respectively) are greater than those of anaplasmosis (35.2% and 0.5%, respectively) [23]. National and NY POWV infection fatality rates are higher than national fatality rates for babesiosis [24,27].

POWV infection incidence peaks in the summer months (May–July) and in November. The summertime peak of POWV infection aligns with other tick-borne disease, as *I. scapularis* nymphs, the developmental stage responsible for most cases of anaplasmosis, Lyme disease, and babesiosis, are most active during the summer months [22–24, 28]. This finding can be attributed to the difficulty of finding and removing nymphal ticks during the period of ≥12–48 h it takes to transmit *Anaplasma phagocytophilum*, *B. burgdorferi*, and *Babesia microti*, and to increased time spent outdoors in tick habitat [28, 29]. While there are minor seasonal peaks in the incidence of other tick-borne pathogens, such as *A. phagocytophilum*, in the fall when adult *I. scapularis* are most active, these pathogens typically require longer attachment times for transmission. In contrast, POWV can be transmitted in as little as 15 min, which reduces the opportunity for tick removal before viral transmission occurs [13, 14]. As a result, adult ticks contribute less to the transmission of most other tick-borne diseases as they are larger, easier to detect, and often removed before infection can occur [30]. The November peak in POWV incidence is thus distinct due to its rapid transmission time, reflecting the virus’s unique transmission dynamics compared with other pathogens.

Over the study period, two of the 44 reported cases of human POWV infection were recorded in infants of just 23 days and 1 month old. Cases of infant POWV infection are speculated to have occurred through contact with a tick carried indoors by a household member, pet, or another potential vehicle. Human contact with cats, dogs, and other household pets is known to increase human tick-borne disease risk [31]. Pet ownership has been shown to significantly increase the risk of finding ticks crawling on or attached to household members when compared to households with no pets [32]. Pet owners should conduct regular and thorough tick checks on all household members, including pets, to minimize risk of infection.

**Figure 5.**
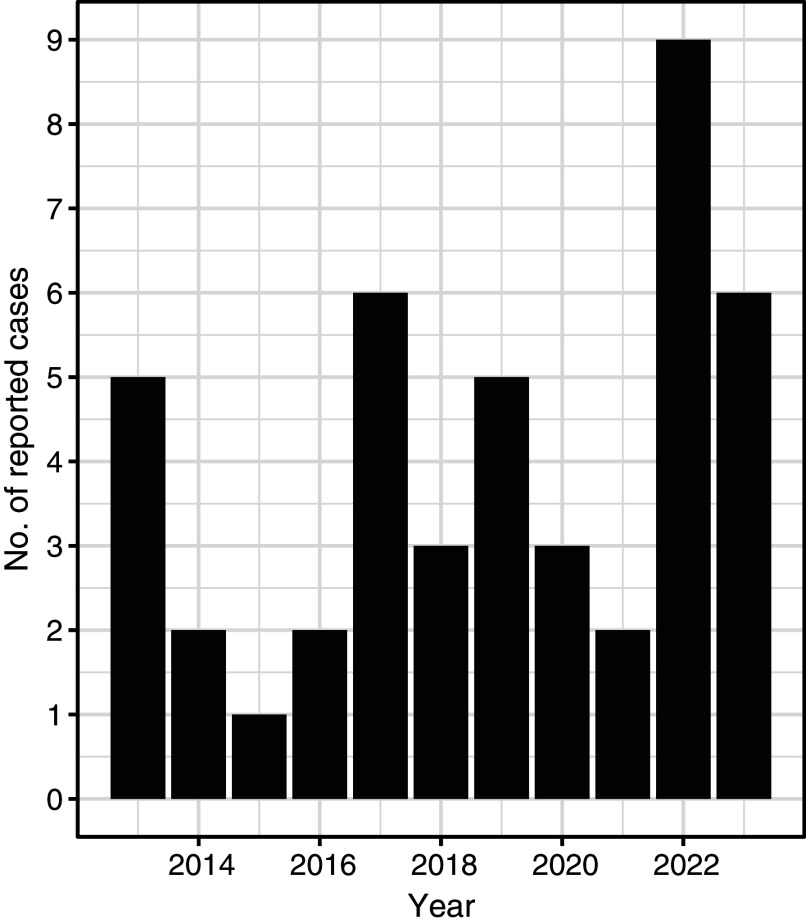
Number of reported cases of Powassan virus infection by year of illness onset, New York, 2013–2023 (N = 44).

Higher POWV infection case numbers typically occurred in odd-numbered years over the study period, apart from 2022, during which the peak number of infections across the study period occurred (Figure 5). *I. scapularis* populations are typically lower across NYS in even-numbered years compared with odd-numbered years due to an *I. scapularis* population crash in 2010 [33]. This biennial pattern is attributable to the 2-year long-life cycle of *I. scapularis* ticks. Possible explanations for the heightened POWV infection incidence in 2022 include the general upward trend of POWV infection incidence in NYS, as well as other environmental and ecological factors that may have bolstered *I. scapularis* populations that year. Notably, precipitation levels across NYS from January to June of 2022 were elevated compared to prior years [34]. Increased early season rainfall may have resulted in favourable conditions for *I. scapularis* survival and questing activity by increasing humidity, maintaining moist leaf litter, and promoting denser vegetation cover [35]. Further, the growing use of commercial tick-testing panels by healthcare providers, which detect antibodies to multiple tick-borne pathogens including POWV, may result in incidental identification of infections. Such cases may occur in individuals who are asymptomatic for POWV or whose symptoms are attributable to coinfection with another *I. scapularis*–associated illness presenting with overlapping generalized symptoms [36, 37]. These incidental detections suggest that a proportion of POWV exposures may be subclinical. Additionally, milder cases of POWV without neurologic involvement may be identified more frequently due to the increased capacity of testing platforms and laboratories performing serologic assays.

To better understand the emergence and distribution of POWV in NYS, it is important to consider the broader ecological and environmental factors influencing its transmission and dispersal. The distribution of *I. scapularis* is associated with the availability of suitable habitat as well as the abundance of preferred hosts such as the white-tailed deer (*Odocoileus virginianus)* [11, 38]. During the 1800s, deforestation and decreases in white-tailed deer populations reduced populations of *I. scapularis* in the Northeastern US [38, 39]. The subsequent rise in POWV disease in the northeastern US, like other *I. scapularis-*borne infections, likely followed the re-establishment of *I. scapularis* ticks after reforestation and the rebound of white-tailed deer populations in the 1900s [12, 38]. These ecological changes may have also increased contact between hosts and the canonical POWV vectors *I. cookei* and *I. marxi.* Overall, vector population growth in NYS has mirrored trends in the Northeast, with substantial increases in *I. scapularis* nymph populations over time, particularly in the southeastern portion of the state [33, 40].

Spatial assessment of the emergence of POWV indicates that the rise in POWV infections as well as prevalence of POWV in tick populations is not occurring uniformly across NYS. Instead, spatial clustering of cases and elevated ERI values demonstrate areas of high-risk in both the Capital and Metropolitan regions of NYS (Figure 4). Like POWV, the emergence of anaplasmosis and babesiosis in NYS have also been focalized, with anaplasmosis occurring primarily in the Capital region and babesiosis primarily in the Metropolitan region [23, 41]. These patterns of emergence are also consistent with the early geographic expansion of Lyme disease, whose early focal area was the southeastern portion of NYS [42].

Even in the absence of strong local statistical significance, the observed spatial clustering of POWV infection cases in the Capital and Metropolitan regions of NYS likely reflect underlying ecological factors and virus transmission dynamics. However, specific factors driving localized emergence of POWV remain poorly defined, highlighting a gap in our understanding of the ecological and environmental factors which sustain POWV.

Additionally, the transmission dynamics of POWV are complex, involving horizontal transmission between ticks and hosts, as well as vertical transmission between adult ticks and their offspring, and co-feeding transmission, where infected ticks feed near uninfected ticks on the same host [43]. These transmission mechanisms can sustain and amplify POWV in localized tick populations, potentially leading to focal areas of high infection prevalence. Explorations of the dispersal history of POWV and its transmission foci suggest that POWV is maintained in highly localized transmission foci, which could in part explain spatial clustering of high ERI values and human POWV infections in NYS [11, 44]. While the relatively low number of confirmed human cases of POWV infection limits statistical power for analyses like the local Moran’s *I*, spatial clustering can still be investigated. The observed number of cases in ecologically suitable areas suggest predictable patterns of virus transmission. These are likely shaped by focal tick infection dynamics, host availability, and additional landscape factors that facilitate human exposure to *I. scapularis* ticks in the Capital and Metropolitan regions of NYS.

Notable limitations in our methodology include host-seeking tick sampling differences by location, differences in vector surveillance activities across NYS regions, repeated sampling at some sites while not at others, and an overall increase in surveillance over the study period. A subset of tick collection sites was also selected based on reported POWV cases, which may introduce bias in local ERI values and observed spatial clustering. While most sites were selected independently based on tick-habitat suitability, we note this as a potential limitation in interpreting spatial clustering results. Additional limitations include the likely underestimation of the incidence of POWV infection due to subclinical and asymptomatic cases which may have been unreported over the study period. The level of awareness of tick-borne disease among health care providers and in the general public likely varies across NYS. Differences in behaviour, diagnosis, and reporting make accurately estimating the burden of POWV infection challenging. The lack of awareness surrounding emerging tick-borne diseases like POWV infection allows for the neglect of co-infection testing by healthcare providers, as well as the potential for undiagnosed cases. This contributes to less accurate case counts and difficulty elucidating the true incidence of POWV infection across NYS.

The assessment of POWV infection epidemiology along with POWV surveillance efforts allows us to identify high-risk populations and anticipate future risk of POWV infection. This enables public health efforts, such as educational messaging, to be targeted toward populations who are at the highest risk. Educational messaging stressing the use of permethrin-treated clothing and gear, appropriate repellent use, carefully checking pets for ticks, and avoidance of tick habitat for those engaging in outdoor activities or living in high-risk areas can help to reduce the risk of POWV infection. Furthermore, messaging can be targeted toward healthcare providers to increase awareness surrounding tick-borne diseases like POWV, with an emphasis on testing for co-infections in patients presenting with tick-borne disease symptoms. This can help us to better understand the overall burden of tick-borne diseases, including POWV infection, and their changing trends in incidence over time.

## Data Availability

The data that support the findings of this study are available from the corresponding author upon reasonable request.

## References

[1] Ebel GD (2010) Update on Powassan virus: Emergence of a north American tick-borne flavivirus. Annual Review of Entomology. 55, 95–110. 10.1146/annurev-ento-112408-085446. 19961325

[2] Gritsun TS, Lashkevich VA and Gould EA (2003) Tick-borne encephalitis. Antiviral Research 57, 129–146. 10.1016/s0166-3542(02)00206-1. 12615309

[3] Centers for Disease Control and Prevention. (2024) Powassan virus. Historic data (2004–2023). https://www.cdc.gov/powassan/data-maps/historic-data.html (accessed 14 November 2024).

[4] McLean DM and Donohue WL (1959) Powassan virus: Isolation of virus from a fatal case of encephalitis. Canadian Medical Association Journal. 80, 708–711. 13652010 PMC1830849

[5] Goldfield M, et al. (1973) A non-fatal human case of Powassan virus encephalitis. The American Journal of Tropical Medicine and Hygiene. 22, 78–81. 10.4269/ajtmh.1973.22.78. 4684890

[6] McMinn RJ, et al. (2023) Phylodynamics of deer tick virus in North America. Virus Evolution. 9, vead008. 10.1093/ve/vead008. 36846826 PMC9943884

[7] Telford SR, et al. (1997) A new tick-borne encephalitis-like virus infecting New England deer ticks, Ixodes dammini. Emerging Infectious Diseases. 3, 165–170. 10.3201/eid0302.970209. 9204297 PMC2627606

[8] Piantadosi A and Solomon IH (2022) Powassan virus encephalitis. Infectious Disease Clinics of North America. 36, 671–688. 10.1016/j.idc.2022.03.003. 36116842 PMC9494578

[9] Artsob H (1989) Powassan encephalitis. In Monath TP (ed), The Arboviruses: Epidemiology and Ecology. Boca Raton: CRC Press, pp. 29–49.

[10] Goethert HK, et al. (2021) Incrimination of shrews as a reservoir for Powassan virus. Communications Biology. 4, 1319. 10.1038/s42003-021-02828-1. 34811484 PMC8608897

[11] Vogels CBF, et al. (2023) Phylogeographic reconstruction of the emergence and spread of Powassan virus in the northeastern United States. Proceedings of the National Academy of Science. 120, e2218012120. 10.1073/pnas.2218012120. PMC10120011 37040418

[12] Eisen RJ, Eisen L and Beard CB (2016) County-scale distribution of Ixodes scapularis and Ixodes pacificus (Acari: Ixodidae) in the continental United States. Journal of Medical Entomology. 53, 349–386. 10.1093/jme/tjv237. 26783367 PMC4844559

[13] Feder HM, et al. (2021) Powassan virus encephalitis following brief attachment of Connecticut deer ticks. Clinical Infectious Diseases. 73, e2350–e2354. 10.1093/cid/ciaa1183. 33111953 PMC8492136

[14] Ebel GD and Kramer LD (2004) Short report: Duration of tick attachment required for transmission of powassan virus by deer ticks. The American Journal of Tropical Medicine and Hygiene. 71, 268–271. 10.4269/ajtmh.2004.71.3.0700268. 15381804

[15] New York State Public Health Law § 2102. Communicable diseases; laboratory reports and records. Albany, NY, USA: New York State Legislature. https://www.nysenate.gov/legislation/laws/PBH/2102. Updated 22 September 2014. (accessed 3 February 2025).

[16] Centers for Disease Control and Prevention (CDC). (2021) Arboviral diseases, neuroinvasive and non-neuroinvasive 2015 case definition. https://ndc.services.cdc.gov/case-definitions/arboviral-diseases-neuroinvasive-and-non-neuroinvasive-2015/ (accessed 14 November 2024).

[17] Prusinski MA, et al. (2014) Prevalence of *Borrelia burgdorferi* (Spirochaetales: Spirochaetaceae), *Anaplasma phagocytophilum* (Rickettsiales: Anaplasmataceae), and *Babesia microti* (Piroplasmida: Babesiidae) in *Ixodes scapularis* (Acari: Ixodidae) collected from recreational lands in the Hudson Valley region, New York state. Journal of Medical Entomology. 51, 226–236. 10.1603/me13101. 24605473

[18] Keirans JE and Clifford CM (1978) The genus ixodes in the United States: A scanning electron microscope study and key to the adults. Journal of Medical Entomology. 2, 1–149. 10.1093/jmedent/15.suppl2.1. 401322

[19] Dupuis AP, II, et al. (2013) Isolation of deer tick virus (Powassan virus, lineage II) from Ixodes scapularis and detection of antibody in vertebrate hosts sampled in the Hudson Valley, New York state. Parasites & Vectors. 6, 185. 10.1186/1756-3305-6-185. 24016533 PMC3711734

[20] United States Census Bureau. (2025) American community survey 5-year data (2009–2023). https://www.census.gov/data/developers/data-sets/acs-5year.html (accessed 23 March 2025).

[21] Mather TN, et al. (1996) Entomologic index for human risk of Lyme disease. American Journal of Epidemiology. 144, 1066–1069. 10.1093/oxfordjournals.aje.a008879. 8942438

[22] Schwartz AM, et al. (2017) Surveillance for Lyme disease — United States, 2008–2015. MMWR Surveillance Summaries. 66, 1–12. 10.15585/mmwr.ss6622a1. PMC5829628 29120995

[23] Russell A, et al. (2021) Epidemiology and spatial emergence of Anaplasmosis, New York, USA, 2010–2018. Emerging Infectious Diseases 27, 2154–2162. 10.3201/eid2708.210133. 34287128 PMC8314826

[24] Gray EB and Herwaldt BL (2019) Babesiosis surveillance — United States, 2011–2015. MMWR Surveillance Summaries 68, 1–11. 10.15585/mmwr.ss6806a1. 31145719

[25] Centers for Disease Control and Prevention (CDC). (2024) Powassan virus. Historic data (2004–2023). https://www.cdc.gov/powassan/data-maps/historic-data.html (accessed 4 January 2025).

[26] Wilson N, et al. (2023) Tick bite risk factors and prevention measures in an area with emerging Powassan virus disease. Public Health Challenges, 2. 10.1002/puh2.136. PMC11118757 38800642

[27] Bloch EM, et al. (2022) Epidemiology of hospitalized patients with babesiosis, United States, 2010–2016. Emerging Infectious Diseases. 28, 354–362. 10.3201/eid2802.210213. 35076004 PMC8798708

[28] Lin S, et al. (2019) The effects of multiyear and seasonal weather factors on incidence of Lyme disease and its vector in New York state. Science of the Total Environment. 665, 1182–1188. 10.1016/j.scitotenv.2019.02.123. 30893749 PMC12191150

[29] Eisen L (2018) Pathogen transmission in relation to duration of attachment by Ixodes scapularis ticks. Ticks and Tick-Borne Diseases. 9, 535–542. 10.1016/j.ttbdis.2018.01.002. 29398603 PMC5857464

[30] Centers for Disease Control and Prevention (CDC).(2024) Lyme disease. How Lyme disease spreads. https://www.cdc.gov/lyme/causes/index.html (accessed 23 March 2025).

[31] Rabinowitz PM, Gordon Z and Odofin L (2007) Pet-related infections. American Academy of Family Physicians. 76, 1314–1322. 18019874

[32] Jones EH, et al. (2017) Pet ownership increases human risk of encountering ticks. Zoonoses and Public Health 65, 74–79. 10.1111/zph.12369. 28631423 PMC7053298

[33] New York State. (n.d.) Nymph deer tick density chart: beginning 2008. https://health.data.ny.gov/Health/Nymph-Deer-Tick-Density-Chart-Beginning-2008/555i-97td (ccessed 24 April 2025).

[34] NOAA National Centers for Environmental Information (NCEI). (2025) Climate at a glance: statewide time series. https://www.ncei.noaa.gov/access/monitoring/climate-at-a-glance/statewide/time-series (accessed 7 May 2025).

[35] Leal B, et al. (2020) Questing by tick larvae (Acari: Ixodidae): A review of the influences that affect off-host survival. Annals of the Entomological Society of America. 113, 425–438. 10.1093/aesa/saaa013. 33244354 PMC7677832

[36] Klontz EH, Chowdhury N and Branda JA (2024) Laboratory testing for Powassan virus: Past, present, and future. The Journal of Infectious Diseases. 230, S70–S75. 10.1093/infdis/jiae197. 39140722

[37] Tokarz R, et al. (2018) A multiplex serologic platform for diagnosis of tick-borne diseases. Scientific Reports 8, 3158. 10.1038/s41598-018-21349-2. 29453420 PMC5816631

[38] Spielman A, et al. (1985) Ecology of Ixodes dammini-borne human babesiosis and Lyme disease. Annual Review of Entomology. 30, 439–460. 10.1146/annurev.en.30.010185.002255. 3882050

[39] Lee X, et al. (2013) Hunter-killed deer surveillance to assess changes in the prevalence and distribution of Ixodes scapularis (Acari: Ixodidae) in Wisconsin. Journal of Medical Entomology. 50, 632–639. 10.1603/me12234. 23802460

[40] Tran T, et al. (2020) Spatio-temporal variation in environmental features predicts the distribution and abundance of *Ixodes scapularis*. International Journal for Parasitology 51, 311–320. 10.1016/j.ijpara.2020.10.002. 33359203 PMC7940570

[41] O’Connor , et al. (2021) A comparative spatial and climate analysis of human granulocytic anaplasmosis and human babesiosis in New York state (2013-2018). Journal of Medical Entomology. 58, 2453–2466. 10.1093/jme/tjab107.40. 34289040 PMC8824452

[42] Chen H, et al. (2005) Epidemic and spatial dynamics of Lyme disease in New York state, 1990-2000. Journal of Medical Entomology. 42, 899–908. 10.1093/jmedent/42.5.899. 16363174

[43] Lange RE, et al. (2024) Direct evidence of Powassan virus vertical transmission in *Ixodes scapularis* in nature. Viruses 16, 456. 10.3390/v16030456. 38543821 PMC10974323

[44] Robich RM, et al. (2024) Prevalence and genetic diversity of deer tick virus (Powassan virus, lineage II) in *Ixodes scapularis* ticks in five habitats at a nature reserve in southern Maine, United States. American Journal of Tropical Medicine and Hygiene. 111, 1311–1319. 10.4269/ajtmh.23-0643. 39406212 PMC11619509

